# Genome-wide analysis-based single nucleotide polymorphism marker sets to identify diverse genotypes in cabbage cultivars (*Brassica oleracea* var. capitata)

**DOI:** 10.1038/s41598-022-24477-y

**Published:** 2022-11-21

**Authors:** Jinkwan Jo, Min-Young Kang, Kyung Seok Kim, Hye Rim Youk, Eun-Jo Shim, Hongsup Kim, Jee-Soo Park, Sung-Chur Sim, Byung Cheon Yu, Jin-Kee Jung

**Affiliations:** 1 Seed Testing and Research Center, Korea Seed & Variety Service, Gimcheon, 39660 Republic of Korea; 2 Department of Pediatrics-Nutrition, Children’s Nutrition Research, Baylor College of Medicine, Houston, TX 77030 USA; 3 Department of Bioresources Engineering, Sejong University, Seoul, 05006 Republic of Korea

**Keywords:** Biotechnology, Molecular biology, Plant sciences

## Abstract

Plant variety protection is essential for breeders’ rights granted by the International Union for the Protection of New Varieties of Plants. Distinctness, uniformity, and stability (DUS) are necessary for new variety registration; to this end, currently, morphological traits are examined, which is time-consuming and laborious. Molecular markers are more effective, accurate, and stable descriptors of DUS. Advancements in next-generation sequencing technology have facilitated genome-wide identification of single nucleotide polymorphisms. Here, we developed a core set of single nucleotide polymorphism markers to identify cabbage varieties and traits of test guidance through clustering using the Fluidigm assay, a high-throughput genotyping system. Core sets of 87, 24, and 10 markers are selected based on a genome-wide association-based approach. All core markers could identify 94 cabbage varieties and determine 17 DUS traits. A genotypes database was validated using the Fluidigm platform for variety identification, population structure analysis, cabbage breeding, and DUS testing for plant cultivar protection.

## Introduction

Cabbage is the fourth most popular vegetable globally. In 2020, the total worldwide production of cabbage, along with other species of *Brassica,* was 70.9 million tons, with import and export values of $2.3 and $1.9 billion, respectively (FAOSTAT). Cabbage is a low-calorie, nutrient-dense vegetable with high concentrations of vitamins, minerals, and antioxidants, which may help prevent heart disease and some types of cancer^1–3^. *Brassica oleracea* (2n = 2x = 18) is an allogamous diploid species with significant inbred depression^4^. Allogamous crops are highly susceptible to the effects of genetic drift, which has a direct impact on breeding and germplasm management^5^. The economic and nutritional aspects, in addition to the mode of cabbage reproduction, have pushed breeders to invest in the development of new cabbage varieties. Thus, plant variety protection (PVP) is crucial because it allows breeders to preserve their investments in new crop variety establishment. PVP was granted per the rules established by the International Union for the Protection of New Varieties of Plants (UPOV)^6^.

Distinctness, uniformity, and stability (DUS), which requires new varieties to be distinct from the reference panel, uniform across seeds that comprise the variety, and stable within the environments, is a critical component of PVP^7^. Current DUS is primarily based on morphological traits, with isozyme polymorphism and molecular markers being used on occasion; however, this is labour-intensive and time-consuming^8^. Because most DUS traits have low heritability, they can be easily influenced by environmental factors, and the inconsistency of DUS traits across scoring organisations is negatively correlated with heritability^9^. Therefore, the use of molecular markers as DUS descriptors has been discussed in UPOV^10,11^. Although the markers and DUS traits should be perfectly co-segregated, UPOV suggests the use of molecular markers^11^. In response to the development of high-throughput genotyping technology, the concept of genomic DUS has been suggested, highlighting the importance of transitioning to a reliable genomics system for registration of new crop varieties^8,9,12,13^.

In the early stage of using molecular markers for DUS, simple sequence repeat (SSR) markers were used, such as 28 SSR markers in maize^14^, 5 SSR markers in rice^15^, 6 SSR markers in tomato^16^ and 11 SSR markers in cabbage^17^. However, SSR markers have some major drawbacks related to the wide use of dinucleotide loci; neighbouring alleles are very close to each other, making their distinction and separation difficult during allele binning^18^. Compared to SSRs, single nucleotide polymorphism (SNP) markers have several advantages, including abundance across the genome, low mutation rates, high reproducibility, and ease of automation for high-throughput and cost-effective genotyping^19,20^. Recent advances in NGS technology have facilitated the discovery of genome-wide SNPs, which are advantageous for plant breeding and genetics^20,21^.

In cabbage, resequencing of *B. oleracea* found a total of 2200 SNPs for the line ’01-88’ and ’02-12’^22^, and transcriptome sequencing of *B. oleracea* found 1167 SNPs for the line ‘C1194’ and ‘C1234’^23^; these identified SNPs were used to construct the respective genetic linkage maps^22,23^. Quantitative trait locus (QTL) analyses were conducted for the 24 main agronomic traits of cabbage using a linkage map of InDel and SSR markers^24,25^. More recently, the genomes of two *B. oleracea* morphotypes, cauliflower and cabbage, were assembled and compared, yielding 120 K high-confidence structural variants (SVs)^26^. Population analysis of 271 *B. oleracea* accessions using these SVs showed that the SVs clearly separated different morphotypes, suggesting that SVs were associated with *B. oleracea* intraspecific divergence^26^. The heterozygosity ranged from 0.47 to 10.52 among the different accessions (median 3.59)^26^.

Despite the abundance of previously discovered SNPs in cabbage, this genomic information has not been successfully applied for variety identification in cultivated cabbage accessions, including in commercial F1 varieties. Here in this study, we detected genome-wide SNPs using genotype-by-sequencing (GBS) on 96 commercial cabbage varieties. We selected and validated 87, 24, and 10 core markers for application to the Fluidigm platform, a high-throughput SNP genotyping system for rapid and high-throughput variety identification. The SNP data generated in this study will be valuable for molecular breeding and genetic analysis, and the developed markers will be suitable for assessing cabbage purity and diversity.

## Results

### Genome-wide SNP mining in cabbage varieties

To mine variants from 96 cabbage varieties, a GBS library was constructed using single *Ape*KI enzyme digestion and sequenced on two lanes of HiSeq 2500. A total of 736,580,268 reads with a length of 74.39 Gb were generated from lane A and lane B. A total of 684,444,768 reads were demultiplexed, with an average of 7,129,633 reads per variety. A total of 171,060,599 demultiplexed forward reads of lane A were used to construct GBS tags for the Tassel 5 GBS v2 pipeline. In total, 16,331,472 tags were generated from 164,480,050 well-barcoded reads. After removing the replicates, 15,087,535 tags were filtered with an average depth of 335 per tag. After alignment with the reference genome, the *B. oleracea* var. oleracea ‘BOL’, a total of 1,070,933 tag tables consisting of 913,054 mapped tags and 157,879 unmapped tags were constructed (Table 1).

**Table 1:** Summary of genotype-by-sequencing (GBS) in cabbage varieties.

Class	No
Number of samples	96
Total number of raw reads	736,580,268
Lane A	368,334,308
Lane B	368,245,960
Total length of reads	74,394,607,068
Total number of demultiplexed reads	684,444,768
Undetermined reads	52,135,500
Average number of reads per variety	7,129,633
Demultiplexed read 1 of lane A	171,060,599
Total number of candidate reads	164,480,050
Total number of tags	16,331,472
Total number of tags without replication	15,087,535
Average depth per each tag	335
Total number of tags mapped	913,054
Tags not mapped	157,879
Size of all tags in tag table	1,070,933
Total number of SNPs MAF > 0.05	218,621
SNPs and missing < 30% for association analysis	57,874
Filtered SNPs missing < 10% for structural analysis	26,301

SNP, single nucleotide polymorphism; MAF, minor allele frequency.

Based on the tag table, a total of 218,621 SNPs were called with the default parameters of the Tassel 5 GBS v5 pipeline, an optional parameter of 75 length of *K*-mer and over 0.05 a minor allele frequency from 96 cabbage varieties. After filtering for less than 30% and 10% missing data, 57,874 SNPs and 26,301 SNPs were filtered, respectively (Table 1). A total of 57,874 SNPs were used to analyse associations with the 19 test guidance (TG) traits of UPOV, which were classified into three types: QL, qualitative; QN, quantitative; and PQ, pseudo-qualitative (Table 2).

**Table 2:** Traits of test guidelines of UPOV for 96 cabbage varieties.

Number	Trait	Unit	Top 200 SNPs		Cluster identification		
			min. −log(P)	max. −log(P)	Marker set	p-value	Group
QL16	Outer leaf: reflexion of margin	Grade (1,9)	2.77	4.86	–	–	–
QL24	Head: reflexion of margin of cover leaf	Grade (1,9)	2.19	3.64	F24	0.0029	1, 5/99
QN01	Plant: height	cm	2.3	5.86	F24	3.E−05	1/3, 99/5
QN02	Plant: maximum diameter	cm	2.26	4.05	F24	3.2E–11	1–3/4–99
QN04	Plant: attitude of outer leaves	Grade (3,5,7)	2.42	4.55	F87	0.019	5, 6, 99
QN05	Outer leaf: size	cm	2.28	4.52	F10	6.E−06	1/5, 6
QN12	Outer leaf: intensity of colour	Grade (1,3,5)	2.22	4.35	F10	6.E−08	5/others
QN15	Outer leaf: undulation of margin	Grade (3,5,7)	2.81	5.16	F87	6.E−04	3–6
QN19	Head: length	cm	2.27	3.78	F24	0.035	4/2, 99
QN20	Head: diameter	cm	2.24	3.43	F87	2.7E−09	1–4/5–99
QN22	Head: cover	Grade (2,3)	2.08	3.89	F24	0.014	3/4, 5
QN27	Head: anthocyanin colouration of cover leaf	Grade (1,3,5,7)	2.66	7.26	F87, F24	< 2.2E−16	1–6/99
QN32	Head: relative length of interior stem compared to length of head	cm	2.27	4.08	F24	0.0057	1, 6/4, 5, 99
PQ06	Outer leaf: shape of blade	Grade (3,4,5)	2.27	4.59	F87	0.0048	3, 5/99
PQ11	Outer leaf: colour (with wax)	Grade (1,2,5)	2.88	8.3	F87, F24	< 2.2E−16	1–6/99
PQ17	Head: shape in longitudinal section	Grade (2,3,4,6,7)	2.2	4.28	F87	0.0073	–
PQ18	Head: shape of base in longitudinal section	Grade (1,2)	2.25	4.33	F87	5.E−06	5–99
PQ25	Head: colour of cover leaf	Grade (1,5)	2.84	8.55	F87, F24	< 2.2E−16	1–6/99
PQ28	Head: internal colour	Grade (2,4)	2.85	7.38	F87, F24	< 2.2E−16	1–6/99

SNP, single nucleotide polymorphism.

The P-values of 57,874 SNPs associated with each of the 19 TG traits were analysed, and 3800 SNPs were chosen from the top 200 ranked SNPs, with P-values ranging from 2.19 to 8.30 for each trait. A total of 2941 SNPs were filtered without replicated positions of SNPs with more than two traits and ranked P-values (Table 3).

**Table 3:** Chromosomal location of SNPs identified in 96 cabbage varieties.

Class	Total SNPs		Filtered SNPs		Candidate SNPs		Core SNPs	
Genic region	129,108	47.9%	1313	44.6%	328	94.3%	84	96.6%
CDS—synonymous	40,202	14.9%	707	24.0%	210	60.3%	48	55.2%
CDS—non-synonymous	30,186	11.2%	489	16.6%	118	33.9%	36	41.4%
Splice region	51,166	19.0%	3	0.1%	0	0.0%	0	0.0%
Intron	7554	2.8%	114	3.9%	0	0.0%	0	0.0%
Intergenic region	140,279	52.1%	1628	55.4%	20	5.7%	3	3.4%
Intergenic	50,770	18.8%	520	17.7%	1	0.3%	0	0.0%
Upstream gene	36,605	13.6%	450	15.3%	8	2.3%	1	1.1%
Downstream gene	33,692	12.5%	422	14.3%	8	2.3%	1	1.1%
Up/Downstream gene	19,212	7.1%	236	8.0%	3	0.9%	1	1.1%
Total	269,387	100%	2941	100%	348	100%	87	100%

SNP, single nucleotide polymorphism; CDS, coding sequence.

**Figure 1:**
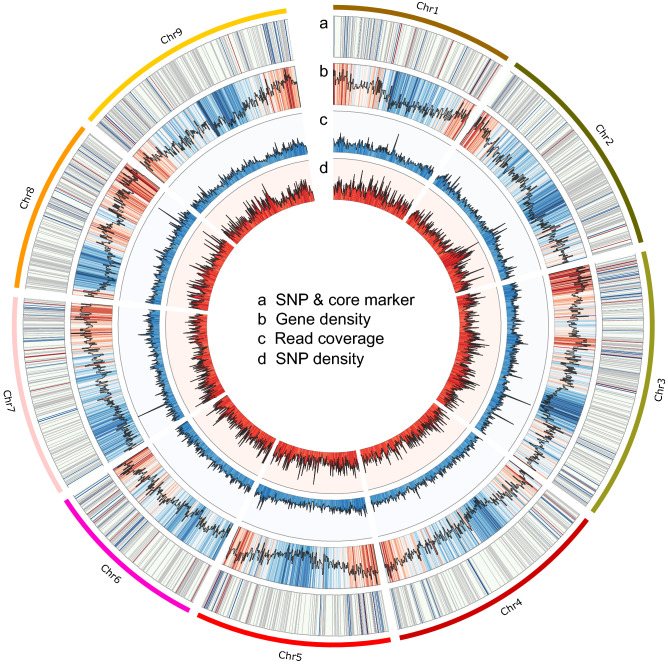
Genomic feature of *Brassica oleracea*. (**a**) Position of single nucleotide polymorphisms (SNPs) and core markers, red and blue colours indicate the position of 87 core markers and 348 candidate markers, respectively. Grey colour indicates 2941 SNPs. (**b**) Gene density of annotation *B. oleracea* var. oleracea ‘BOL’ release 41, red and blue indicate gene-rich and gene-poor regions, respectively. (**c**) Read coverage of genotype-by-sequencing (GBS); deep blue indicates over 75% and light blue indicates less than 25%. (**d**) SNP density, Deep red indicates over 75% and light red indicates less than 25%.

These 2941 SNPs were distributed along the eight cabbage chromosomes, ranging from 256 to 433 SNPs per chromosome (Fig. 1, Supplementary Table 1). A subset of 348 candidate SNPs was chosen based on the genomic region of annotation of the *B. oleracea* genome ‘BOL’ release 41 or their distribution of genome (Table 3, Fig. 1).

Out of the 218,621 SNPs identified, 140,279 SNPs (52.1%) and 129,108 (47.9%) were located in the intergenic and genic regions, respectively, of which 70,388 (26.1%) were located in exons and 7554 (2.8%) in introns (Table 3). Out of the 2941 SNPs filtered, 1628 (55.4%) and 1313 (44.6%) were located in the intergenic and genic regions, respectively. Of the 348 candidate SNPs, 210 (60.3%) were discovered to be synonymous SNPs, and 118 (33.9%) were discovered to be non-synonymous SNPs, and 20 (5.7%) were located in the intergenic region (Table 3).

### SNP chip-based genotyping with core SNP marker sets and genomic diversity of cabbage varieties

To develop SNP markers for efficient variety identification, we selected 348 candidate SNPs that had a polymorphic informative value averaged 0.33, ranging from 0.11 to 0.37, a call rate averaged 91.9%, ranging from 72 to 100%, and minor allele frequency averaged 15%, ranging from 1 to 30%. A total of 384 primer sets for the Fluidigm assay were designed using the 348 candidate SNPs. Genotyping was conducted for 94 cabbage varieties with two replicates of no template control using Fluidigm assay with a 99.53% of the genotyping rate.

**Figure 2:**
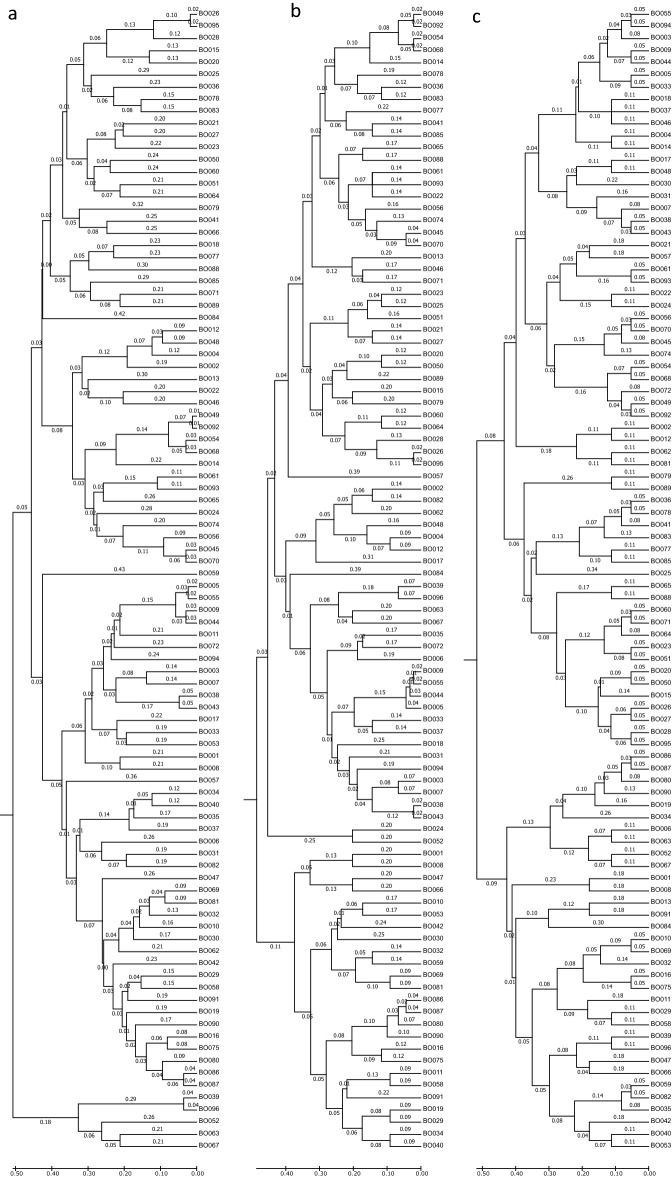
Phylogenetic trees based on Fluidigm genotyping results using core single nucleotide polymorphism (SNP) markers in 94 cabbage varieties. To determine the genetic relationship of cabbage varieties, a total of 94 varieties were genotyped with 87, 24, and 10 core SNP markers. The optimal tree with the sum of branch length is 20.9, 18.6, and 16.5 respectively.

To develop highly accurate core marker sets, additional genotyping was carried out with 96 primer sets that had clearly separated genotypes which were polymorphic and precise (Supplementary Fig. 2). We could identify 94 samples in the previous genotyping of 384 primer sets. Among the 96 primer sets, 87 core markers showed stable and clear genotypes among the genotyping replicates, and these were selected for downstream analyses. Nine markers were excluded as they showed inconsistent genotypes and unusual clustering patterns of genotypes. The 87 core markers accurately identified all 94 cabbage varieties (Fig. 2). The genotype results of all experiments were merged, and a database of 8177 out of 8178 genotypes was generated from the core markers and 94 samples that could be used as a ‘reference variety’ (Supplementary Table 2).

To minimise the number of markers while retaining the performance of variety identification, subsets of 87 core markers were randomly selected based on the uniqueness of the genotype across 94 varieties. The markers were then purposefully included and excluded by the population structure of subsets for evenly distributed across the traits, improving identification of DUS traits. A phylogenetic tree was constructed using the UPGMA method with 87, 24, and 10 subsets of the core markers (Fig. 2). The optimal trees with the sum of branch lengths were 20.9, 18.6, and 16.5, respectively. The minimum length between the most similar varieties increased by 0.01, 0.02, and 0.05, respectively. As the number of markers decreased, the sensitivity of dissimilarity increased. These core sets clearly identified all 94 cabbage varieties, and the number of markers was suitable for efficient genotyping systems such as the IFC platforms of Fluidigm genotyping assays. Therefore, a set of 10 markers would be sufficient to identify varieties and genotyping results, and the primer sequences are provided in Table 4 and Supplementary Table 3.

**Table 4:** Twenty-four core markers used for Fluidigm assay.

Marker	Chr	Position	Allele	MAF	PIC	Variant type	Gene ID	ASP1_SEQ	ASP2_SEQ	LSP_SEQ	STA_SEQ
CQN01-1	C1	16912365	T/A	0.38	0.36	Missense	Bo1g057020	GCAAGGCGGCTAGCGAT	GCAAGGCGGCTAGCGAA	GCGACATGATGGCTTCCACG	CTCAAAGATCGATGAGCTTAAATGC
CQN19-7*	C1	30133135	A/T	0.41	0.37	Missense	Bo1g101450	CCGCAGCCACATGAGTCA	CCGCAGCCACATGAGTCT	ACGCACCACCCCTCTGG	GCTTCGTCGGACCGGA
CQN32-10	C1	40348257	A/C	0.3	0.33	Synonymous	Bo1g141130	GAGATACAGCTGCTAGGTTGACT	AGATACAGCTGCTAGGTTGACG	GAGCCTCCAAGGGAGACGA	CAGAAGCTTGTGTACGTGTTGA
CQN12-2	C1	42832899	T/A	0.27	0.32	Missense	Bo1g153330	CCACCGCCTCCTTGTTTGT	CCACCGCCTCCTTGTTTGA	GGGCTTTCCTGTGCAGCAA	TGTGAGGAGTATGATCCGACC
CQN20-9	C2	2493581	T/G	0.33	0.34	Synonymous	Bo2g010440	TCTCATGGAATGCGATAACGGT	TCTCATGGAATGCGATAACGGG	GCAAACCAGTCATGGCGAGA	CCATAATAAAGACAGTCTTGCTCTCA
CQN22-15	C2	21153749	A/T	0.37	0.36	Missense	Bo2g075400	GGTCGTTTCCGTACCATCCA	GGTCGTTTCCGTACCATCCT	CGTACCAAGATATAGAGCGAGGACG	GCTCTATAGCGGACTCATTTCTTG
CQN02-11*	C2	41871386	G/T	0.24	0.3	Missense	Bo2g134070	CATAGGCTTCGTTGTCCCTAGT	ATAGGCTTCGTTGTCCCTAGC	GGGAAGTTTCTGTGTGCAGCT	TCATAATCAAGGAGCTCCTCCTC
CPQ25-11	C2	47959189	A/C	0.43	0.37	Missense	Bo2g151870	TCAAGCTGGCTTCCTTTATATGGAAA	CAAGCTGGCTTCCTTTATATGGAAC	GTGGCTTCCGTTGTTGCTCT	TTCCAAGGAAGTGAATCTTACACG
CPQ18-17	C2	51429603	C/A	0.09	0.15	Synonymous	Bo2g165400	CGTCAGTCCCTGCACCC	CGTCAGTCCCTGCACCA	CTCCAGCTCCTACGTAGGCA	TTTGGAGCTGTGTTTGCGT
CPQ18-18*	C3	1728366	A/G	0.28	0.32	Missense	Bo3g004620	GTTGCTGCCCTTGTTGGTATTT	GTTGCTGCCCTTGTTGGTATTC	TCCTTCGCCACTGAGAAGCA	GGGTTCATCTTCACTTGCTTTG
CQL24-5	C3	1728375	T/C	0.28	0.32	Missense	Bo3g004620	CTTGCTTTGTTGCTGCCCTTA	TGCTTTGTTGCTGCCCTTG	CCACTGAGAAGCAGTGTAGGC	GTCTTGCGGGTTCATCTTCAC
CPQ17-13	C3	16381523	G/C	0.41	0.37	Synonymous	Bo3g040220	GCACCTACTTCCTCCGCG	GCACCTACTTCCTCCGCC	GCAGGAGTGGTGGTTTTTTCCA	ACTTTTGGTGGCATTTCGGT
CQN02-23	C3	21935944	T/G	0.42	0.37	Missense	Bo3g055490	GGAAAGATCTCAGAGCTCTTAAGCAA	GGAAAGATCTCAGAGCTCTTAAGCAC	TGCTGCGTCTTCTCCATAGCC	CAGTTGGAGTTCAAAAAGGCG
CPQ11-18	C3	34450549	C/G	0.33	0.34	Missense	Bo3g093990	GCTCCGAGCCTACTTCTCAAG	GCTCCGAGCCTACTTCTCAAC	CGATGATGACCGTGAGGGTCT	ACTTCGTCCCGGAGCAG
CPQ11-20*	C3	58838243	T/C	0.33	0.35	Synonymous	Bo3g166990	CGAAAGAGAACTCTGAGAAAAACGGA	GAAAGAGAACTCTGAGAAAAACGGG	CTGGACTCGATAACGGCGGA	GGCTGCTTTTAGGTCGGC
CPQ6-30*	C5	6622086	G/C	0.4	0.36	Synonymous	Bo5g019690	GGCTTGAGATTCAGCATCTCTACC	GGCTTGAGATTCAGCATCTCTACG	CCCTTCACATAGCTGCAGCTT	GAGTTTTTTCGCAAAAGACGGC
CQN22-50*	C5	42891943	G/C	0.36	0.35	Missense	Bo5g137520	AGTTGCAAAAGGCTAAAGAGTTTCTTC	AGTTGCAAAAGGCTAAAGAGTTTCTTG	CCGTCGTGAAACTGTGCGT	ACAAGTCGAGGGAGAAGCA
CQN15-28	C5	46259901	T/A	0.36	0.36	Missense	Bo5g150300	AGCCATTATCGGATCCAAACACT	AGCCATTATCGGATCCAAACACA	ACCAAGGAGCAGCTTATGGGA	CATTGAGCCAGCCAGAACA
CQN01-49*	C6	4834902	A/C	0.21	0.28	Synonymous	Bo6g022330	TTAGAAACGGTGAAGCCTTAGCT	GAAACGGTGAAGCCTTAGCG	CAGGCATGTGGTGAAGCACAA	GCTGCACGGAAGTTGAGA
CQL16-30*	C6	37280236	T/A	0.41	0.37	Synonymous	Bo6g119200	ACAGCTCAGAGCTCCTCCT	ACAGCTCAGAGCTCCTCCA	GTCGAAGTTGCAGCCGGTAC	ATCCAGGCGCTTCCGTA
CPQ25-55	C7	20974235	T/C	0.11	0.18	Up/downstream_gene	Bo7g057720	GATCAAGTCGATCACCTCTCGT	ATCAAGTCGATCACCTCTCGC	GTTCGTGAACCCGACGGTG	AGCAGCGATAAAGACTCGGA
CQN04-34*	C7	32444765	T/C	0.26	0.31	Synonymous	Bo7g082370	CGTTCCGACGAAGTTACAGAGT	CGTTCCGACGAAGTTACAGAGC	CTGAACACGGCTTGGATTCTCA	CGGACAGTGGCTTCGC
CQN12-40	C7	43517964	G/A	0.27	0.31	Synonymous	Bo7g109870	GGGTGGTATAGTCTCTGGCTTC	GGGTGGTATAGTCTCTGGCTTT	CCCCATTCGTCTTCCGTCTCT	TGTCGTCTCGCGGAATGTAG
CPQ17-44*	C9	4150485	G/A	0.33	0.34	Synonymous	Bo9g014040	GACCGTCTCAGAAGTACCACTTAAC	GACCGTCTCAGAAGTACCACTTAAT	AGAGCTGCCACTTCATCATGCT	AGGGACAAGATGATTCCAATCAATG

*Asterisk indicates F10 core markers.

Chr, chromosome; PIC, polymorphic information content.

**Figure 3:**
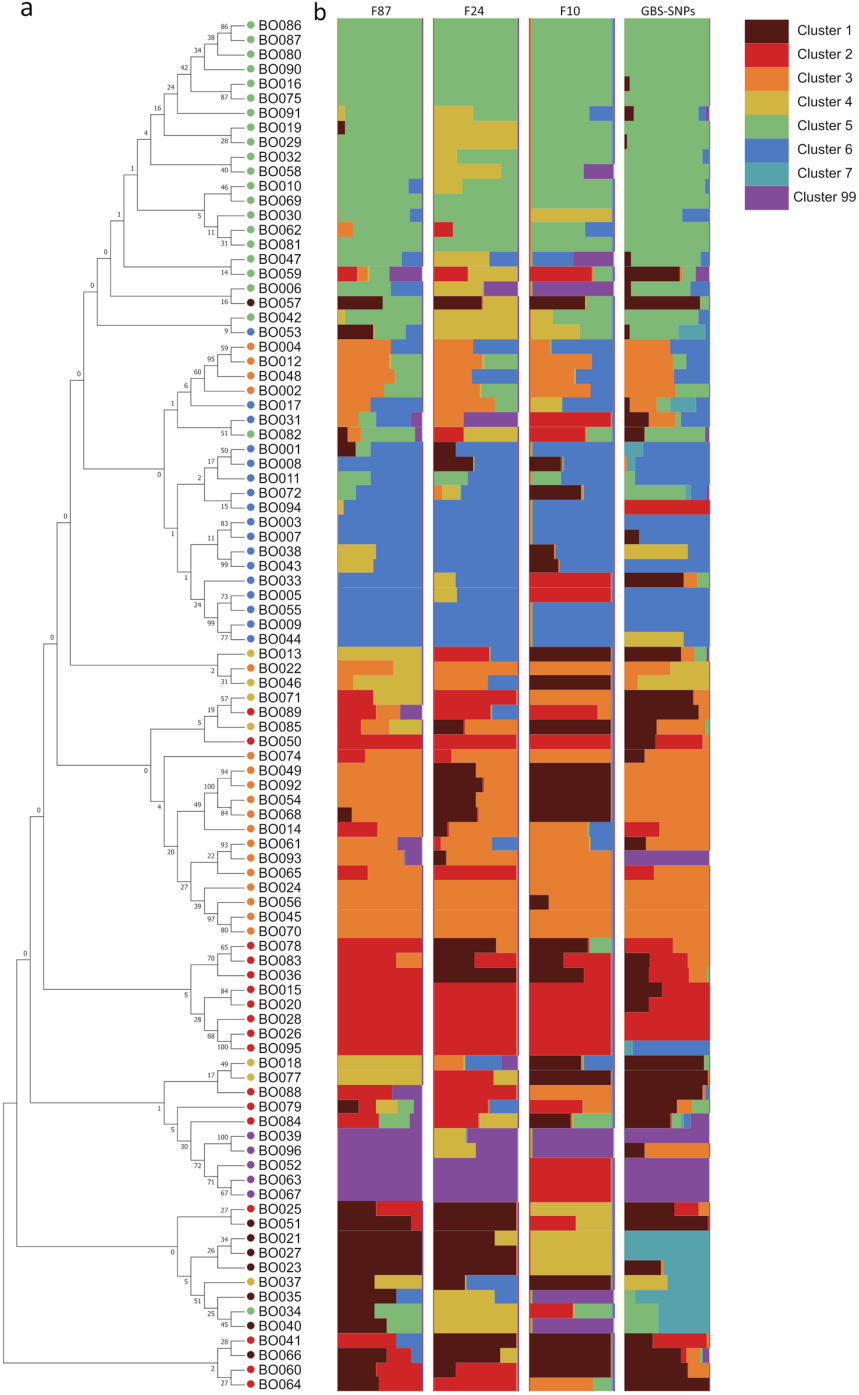
Phylogenetic tree and population structure analysis of 94 cabbage varieties. (**a**) Phylogenetic tree constructed using the neighbour-joining method from genotypes of 87 core markers. Coloured dots indicate consensus of clusters of F87, F24 and F10. (**b**) Q plots for the respective F87, F24, F10, and GBS-SNPs. Each cluster colour indicates a proportion of the q-values.

Principal component analysis (PCA) was performed using 26,301 filtered SNPs. First two PC explained 19.14% of the total variance, indicating that the 96 varieties were clustered into four groups (Supplementary Fig. 1). A phylogenetic tree was constructed for 94 varieties with 87 core markers using the neighbor-joining method with a bootstrap value of 1000 (Fig. 3).

Population structure analysis was conducted using 26,301 GBS-driven SNPs (GBS-SNPs), and Fluidigm tested 87, 24, and 10 core markers (F87, F24, and F10, respectively) (Fig. 3). Marginal likelihoods were maximised for GBS-SNPs and F87 at model complexities of 8 and 7, respectively, and structure plots were chosen at these model complexity levels. Additionally, the F87, F24, and F10 subsets of the 87 core markers were chosen for model complexity 7. Then, 94 cabbage varieties were grouped into clusters and classified using Q plots for each structure (Fig. 3, Supplementary Table 4).

To determine the informative clustering of core marker sets for each trait, Duncan’s new multiple range test (DMRT) was performed between clusters of each trait (Supplementary Table 5). We analysed the association of 19 traits of the UPOV test guidelines with clusters of GBS and Fluidigm markers (Supplementary Table 5). The 18 traits, except for QL16, showed significant differences by cluster (Table 2). The trait of QL16 was not identified by clustering and showed no significant differences between the clusters, with the P-value ranging from 0.5 to 0.92 (Supplementary Table 4). However, PQ17 showed considerable differences with the p-value ranging from 0.0041 to 0.15, but there were no clusters which could identify traits (Table 2, Supplementary Table 5).

**Figure 4:**
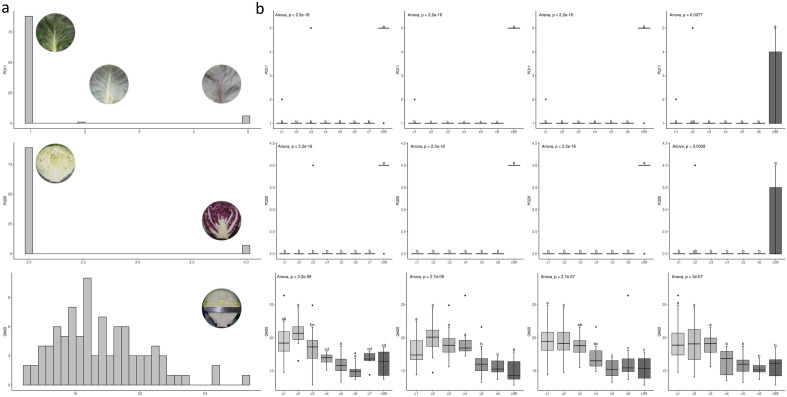
Test guidance (TG) analysis based on cluster of genotype-by-sequencing (GBS) and core sets of Fluidigm markers. (**a**) histogram of phenotypes of PQ11 (Outer leaf: colour), PQ28 (Head: internal colour) and QN20 (Head: diameter). (**b**) Duncan's new multiple range test and boxplot of each cluster based on GBS-SNPs and Fluidigm core markers. Different lowercase letters indicate significant differences between means at p-value < 0.05. SNPs, single nucleotide polymorphisms.

The five traits, PQ11, 17, QN20, 30, and 34, are essential criteria for establishing a new cabbage variety and selecting a ‘reference variety’. PQ11 and QN20 were chosen as representative types of expression among the five traits, and PQ28 was also chosen because of a strong positive correlation (0.995) between PQ11 and PQ28: outer leaf colour and internal colour of the head, respectively (Supplementary Table 6). The phenotypic distributions of the three traits are shown in Fig. 4. The PQ11 and PQ28 were distributed according to three and two criteria, respectively. The distribution of QN20 was as follows: the diameter of the head was between 12 and 27 cm (Fig. 4a). In the case of PQ11 and PQ28, the very strongly positively correlated traits, the clusters of GBS, F87, and F24 showed extremely significant differences (p-value < 2.2e−15). In contrast, F10 showed a highly significant difference (p > 0.005) (Supplementary Table 5). For QN20, the clusters of all markers showed extremely significant differences (p ≤ 2e−07) (Supplementary Table 5).

Ninety-six cabbage varieties were classified into eight clusters (1–7 and 99) using 26,301 GBS-SNPs based on structure Q plots with a model complexity of 8 (Fig. 3, Supplementary Table 4). In the GBS-SNP clusters, 16 out of 19 traits were explained by one or two groups of clusters, of which 13 traits were explained by two groups, and three traits were explained by one group (Supplementary Table 5). Two out of 13 explained traits, PQ11 and PQ28, were explained by the classification of ‘white’ and ‘purple’ groups (Supplementary Table 5). None of the cabbage varieties in the ‘white’ group (clusters 1–7), except the purple cabbage ‘BO096’, showed purple colour. All cabbage varieties in the ‘purple’ group (cluster 99), except the white cabbage ‘BO093’, showed purple colour (Fig. 4b, Supplementary Table 4). Additionally, QN20 was also explained by the classification of ‘big’ and ‘small’ groups (Supplementary Table 5). All cabbage varieties showing the biggest diameter of head were in the ‘big’ group (cluster 2) with the different lowercase letter ‘a’, and all cabbage varieties having the smallest diameter of head in the ‘small’ group (clusters 5 and 6) with the different lowercase letter ‘d’ (Fig. 4b, Supplementary Table 5).

Among the 96 cabbage varieties, 94 were classified into 7 clusters (1–6 and 99) using F87, F24, and F10 (Fig. 3, Supplementary Table 4). In the F87 clusters, 16 out of 19 traits were explained by one or two groups of clusters, where 11 traits were explained by two groups, and five traits were explained by one group (Supplementary Table 4). In the F24 clusters, 17 out of 19 traits were explained by one, two, or three groups of clusters. Two traits were explained by three groups, 11 traits by two groups, and four traits by one group (Supplementary Table 4). In the F10 cluster, 16 out of the 19 traits were explained by one or two groups of clusters. Six traits were explained by the two groups, and 10 traits were explained by one group (Supplementary Table 4).

In the core markers, the two traits, PQ11 and PQ28, were also explained by the ‘white’ and ‘purple’ groups (Fig. 4b, Supplementary Table 5). The F87 clusters perfectly identified the two traits of the samples. None of the cabbage varieties in the ‘white’ group (clusters 1–6) and all cabbage varieties in the ‘purple’ group (cluster 99) showed purple colour. In the clusters of F24, the exception of the white cabbage ‘BO031’ was classified into cluster 99 as a purple group (Supplementary Table 4). In the F10 clusters, the traits could not be identified by grouping. Additionally, QN20 was also explained by two groups, ‘big’ and ‘small’ groups. In the clusters of F87, the big group (a) comprised clusters 1–4, and the ‘small’ group (b) comprised clusters 5, 6, and 99. In the clusters of F24, the big group (a) was clusters 1 and 2, the small group (c) was clusters 5, 6, and 99, and clusters 3 (ab) and 4 (bc) were unclear. In the F10 clusters, the big group (a) comprised clusters 1–3, and the smaller group (b) comprised clusters 4–6 and 99 (Fig. 4b, Supplementary Table 5).

Therefore, in the case of PQ11 and PQ28, the grouping of the GBS clusters did not match the two samples. The core marker sets F87 were perfectly matched, and F24 had a single sample that did not match. In the case of QN20, clusters of GBS could identify the diameter of the head for each cluster with high resolution for clusters 2, 6, and 7. Clusters F89 and F10 identified the diameter of the head for all varieties as two groups, ‘big’ and ‘small’ (Fig. 4b). Therefore, the grouping accuracy in PQ11 and PQ28 increased in core markers compared to GBS-SNPs. Despite a significant decrease in the number of markers, fewer core markers than GBS-SNPs could explain all cabbage varieties as two distinct groups.

Overall, the grouping of clusters using GBS-SNPs, F87, and F10 explained 16 out of the 19 traits. Adding trait QN19, the grouping of clusters using F24 explained 17 out of 19 traits. The number of traits that could be explained by grouping was increased using core markers rather than GBS-SNPs (Supplementary Table 5).

The most informative groups of clusters are listed according to marker sets in Table 2, with the best p-value for TG traits. The clusters of F87 identified 10 traits of QN 4, 15, 20, 27, and PQ 6, 11, 17, 18, 25, 28. The clusters of F24 identified 10 traits of QL 24, QN 1, 2, 19, 22, 27, 32, and PQ 11, 25, 28 with the best p-values among the subsets of core markers. The F10 clusters identified two traits of QN 5, 12 with the best p-value among the subsets of core markers. The trait clusters were classified into one, two, or three groups. The clusters of QN4, 15, and PQ18 could determine one specific trait. The clusters of other traits were classified into two groups, except for QN01, which was classified into three groups (Table 2).

Previously, QTL analysis of cabbage was carried out for 24 major agronomic traits, revealing new QTL clusters in seven regions^24,25^. The seven DUS traits observed in this study were likely connected to seven of the 24 traits in the previous study. We discovered that seven core markers were co-located in six of the seven QTL cluster regions that were supposed to have the most influence on the cabbage phenotype within 1 Mb flanking regions^25^. Of these, three markers were directly linked to previous agronomic QTLs in this region. The two core markers QN01-1 and QN20-5, which are associated with traits, plant height, and head diameter, respectively, were found in region 1 on chromosome 1, which contains the QTLs associated with these traits. The marker QN27-45 linked to the trait ‘Head: anthocyanin colouration of the cover leaf’ was found in region 5 on chromosome 6, which also contained the QTL linked to the trait colour of the head (Table 2).

Upon clustering the cabbage varieties, the core markers could determine 17 traits. F24 could determine the cluster of the cabbage trait PQ11, which is an important criterion for establishing new varieties. F10, a subset of F24, could determine the cluster of the cabbage trait QN20, which is another important criterion to establish new varieties, into two groups with a p-value of 2e−07 (Table 2). Finally, F10 is sufficient for variety identification, F24 is sufficient for both variety identification and determination of the clusters to find the ‘reference variety’ for efficient DUS tests, and F87 provides more precise clusters of traits. Additionally, the three markers discovered in the comparison of previous QTL studies among the core sets will be helpful for cabbage breeding.

## Discussion

We developed a core set of SNP markers to identify cabbage varieties and used these markers to identify TG traits through clustering using a high-throughput genotyping system. In this study, the GBS tag database, a total of 1,070,933 tag tables, was constructed for the GBS v2 pipeline. The filtered 26,301 SNPs mined from the tag database successfully distinguished 96 commercial cabbage varieties cultivated in the Republic of Korea. A total of 2941 SNPs, with the top 200 ranked by the P-value of GWAS (genome-wide association studies) for each TG trait without replicated SNP position, were converted to 348 candidate SNPs for the Fluidigm assay.

Highly accurate core marker sets (F87) were selected from 348 candidate SNPs and generated a database of 8177 genotypes for 94 samples, which could be used as a ‘reference variety’. The population structure analysis using GBS-SNPs classified 96 varieties into eight clusters, and the core sets, F87, F24, and F10, classified 94 varieties into seven clusters. The core markers successfully distinguished 94 commercial cabbage varieties. We found an association between TG traits and GBS and Fluidigm marker clusters. The number of explained traits increased, and the cluster groups became more precise in the clusters of core markers than in GBS-SNPs. These results demonstrate that selecting candidate SNPs based on GWAS is more informative for TG traits while developing core markers.

Linkage disequilibrium (LD) analysis performed for 57,874 SNPs in 96 commercial cabbage varieties revealed that LD decayed within 14 kb in cabbage varieties. Similarly, LD decays within 20 kb in *B. oleracea* broccoli cultivars, whereas 2 kb in *B. oleracea* broccoli landraces and 0.5 kb in *B. oleracea* collard landraces^27,28^. The power of GWAS is dependent on genetic diversity^29^. The magnitude of LD and its decrease with genetic distance plays a crucial role in determining the mapping resolution^30^. The recombination rate influences the rate of LD decay in a population over time^31^. Natural populations, which have historically accumulated events of recombination, are better suited for association mapping than commercial cabbage varieties, with LD that covers long distances. For this reason, we expect that significant SNP markers will seldom be detected over the threshold.

However, clustering of cabbage varieties using core markers successfully determined the traits of cabbage varieties. The core markers were preferred SNPs in the genic region; they have greater stability than other intergenic regions because of the slow mutation rate, promising stable identification of variety, and higher chances of change in the protein structure that might be associated with phenotypic differences between varieties^12,32^. Functional SNPs in genes affect plant phenotypes^33^. For various crops, the GWAS-based approach was used to identify DUS traits associated with SNPs^6,9,13^. Fixed-effects meta-analysis is the most popular method for synthesising GWAS data and is the most effective method for identifying and prioritising phenotype-associated SNPs^34^. Genomic prediction accuracy can be significantly improved by excluding markers that have weak associations with the trait of interest^35^. The accuracy of marker-assisted selection (MAS) increased when there were four or more markers in the haplotype surrounding the QTL^36^. Therefore, the ability of core markers to identify traits of cabbage varieties through clustering may be the result of a GWAS-based approach that provides trait-linked markers and the stacking of trait-linked markers in core marker sets.

Previously, core markers to identify varieties of the PVP system have been developed for various crops^16,37–39^, and genetic markers have been used to predict DUS traits in various crops^6,9,13–15^. To achieve the goals of variety identification and DUS trait prediction, we minimised the number of markers based on phylogenetic analyses. We clustered the cabbage varieties based on population structure analyses to find an association between the limited number of markers and traits. Our previous study specified core markers for commercial lettuce; however, only lettuce type could be identified with its variety^40^. In this study, 17 DUS traits were identified with 100% variety identification. Even among the 17 traits, there are two important traits for the registration of variety. Furthermore, 87 core markers were tested and selected from 384 primers used in the Fluidigm assay. The experiments were carried out using 384, 96, and 87 assays. This process included selecting the confident genotype in replications and the clear genotype, which displayed clear clustering patterns. The genotype database, which contained genotypes from 94 samples, can potentially be used as a database of reference varieties. Therefore, these core marker sets are efficient tools for DUS testing, supporting the selection of ‘reference variety’ within clusters defined by genotyping.

In this study, core markers F87, F24, and F10 correctly identified all 94 cabbage varieties and were suitable for an efficient genotyping system using the IFC platforms of Fluidigm genotyping assays. Specifically, F24 is sufficient for both variety identification and determining the clusters to find the ‘reference variety’ for efficient DUS tests; thus, the number of F24 is suitable for the 192.24 IFC platform, which enables to identify the number of cabbages varieties expanded by 192 samples. In addition, the minimum core marker F10 can be directly converted to a cost-effective gel-based marker system that requires a basic PCR machine and electrophoresis.

Despite the difficulty of mapping due to rare recombination events in the commercial cabbage varieties^41,42^, core markers are related to agronomic QTLs in previous studies, and three directly related markers, QN01-1, QN20-5, and QN27-45, were found. We believe that this is the key to identifying DUS traits and that our core markers and these three markers will be extremely useful for MAS in cabbage breeding.

Finally, the Fluidigm system and genome-wide SNP marker discovery via GBS were successfully used to assess the genetic diversity of cabbage, and the selected core sets of markers were validated using the GWAS approach for variety identification, followed by clustering for DUS trait identification as part of the DUS test of cabbage. We created an SNP database for cabbage variety identification based on the genotyping results of commercial cabbage varieties.

The core markers identified in this study have an accurate genotype, stable variant, powerful platform, and scalability for low-tech laboratories. The constructed SNP database will support cultivar identification, population structure analysis, cabbage breeding, and DUS testing for plant cultivar protection and enforcement of breeder rights. The genome-wide association studies were conducted on F1 cultivars, which prevent identifying the significant SNP markers above the threshold. Therefore, to find and validate the significantly associated core markers that were discovered to be in the previous QTL region, SNP genotyping should be applied to more cabbage varieties, such as bi-parental populations, natural populations and cultivars worldwide. This will help identify diverse cultivars worldwide and increase the usefulness of our SNP databases.

## Methods

### Plant materials and DNA extraction

Ninety-six commercial cabbage varieties were used to obtain genome-wide SNP data for cabbage cultivars. The TG traits were phenotyped according to the UPOV TG/48/7 guidelines for cabbage by the Korea Seed & Variety Service (KSVS, Gimcheon, Korea)^43^. The plants were grown in an open field at the KSVS. We complied guidelines and legislation of the International Union for the Protection of New Varieties of Plants (UPOV) and Ministry of Agriculture, Food and Rural Affairs (MAFRA, Sejong, Korea). Young leaf tissue was collected and 100 mg was measured into a 2 ml microtube. The genomic DNA from each cabbage cultivar was extracted using a modified SDS method ^44^. DNA was quantified using a DS-11 spectrophotometer (Denovix, Inc., Willmington, DE, USA) and qualified using 1.2% agarose gel electrophoresis at 100 V for 30 min.

### Genotype-by-sequencing

The double stranded DNA was quantified for the GBS library using the Quanti-it Picogreen dsDNA Assays Kit (Thermo Fisher Scientific, Inc., Waltham, MA, USA) according to the manufacturer's instructions. To ensure the quality of the DNA for enzyme inhibition, 50 ng of dsDNA from each cabbage cultivar was digested by 1 unit of *Eco*RI at 2 h. A library was constructed for the 96 cabbage varieties at the Institute for Genomic Diversity (Ithaca, NY, USA), following the protocols described by Elshire et al.^45^. The genomic DNA of each variety was single-digested with *Ape*KI and ligated using 96 unique barcodes. The GBS library was sequenced using Hiseq 2500 (Illumina, Inc., San Diego, CA, USA). Tassel 5 GBS v2 pipeline^46^ were used to call SNPs from the sequenced GBS library. The plugin options of pipeline were as follows: QseqToTagCountPlugin, FastqToTagCountPlugin, QseqToTBTPlugin, FastqToTBTPlugin, and MergeTagsByTaxaFilesPlugin: -s 300000000, TagsToSNPByAlignmentPlugin: -mxSites 1000000, MergeDuplicateSNPsPlugin: -callHets, tbt2vcfPlugin: -mnMAF 0.05, -mnLCov 0.1, GBSHapMapFiltersPlugin: -mnSCov 0.8, and GBSHapMapFiltersPlugin: -mnF -mnMAF 0.01 -hLD. The tags were aligned to the reference genome *B. oleracea* var. oleracea ‘BOL’^47^.

### SNP analysis

VCFtools v0.1.10 was used to compile and filter data and prepare the input files for PLINK v1.90. BEDtools v2.30.0 was used to summarise the gene density, read coverage, and SNP density. Analyses were visualised using Circos v0.69-9. The association between genome-wide SNPs and TG traits was calculated using TASSEL with the generalised linear model^48^.

### SNP assessment and genotyping

Candidate SNPs chosen from the GBS data with flanking sequences were validated by Sanger sequencing. The candidate SNPs were converted into SNP type assays using the D3 Assay Design program (Fluidigm Corporation, San Francisco, CA, United States) to be used in Fluidigm 192.24 or 96.96 dynamic arrays. Primer sequences of the markers are listed in Supplementary Table 7. The candidate SNPs were validated using a high-throughput Fluidigm Juno™ system (Fluidigm Corporation, San Francisco, CA, United States) with 94 cabbage varieties, according to the manufacturer’s instructions. SNP type tests were carried out using the 96.96 IFC in accordance with the manufacturer's instructions^49^. The template DNAs were pre-amplified using a pool of locus-specific primer (LSP) and specific target amplification (STA) primers for all target loci, and the amplicons of each variety for all target loci were amplified using a pair of allele-specific primers (ASP). Thereafter, according to the manufacturer’s instructions, fluorescence was measured using the Fluidigm EP1 (Fluidigm Corporation, San Francisco, CA, United States). Further, the SNPs were called using the Fluidigm SNP Genotyping Analysis software v4.5.1. The genotype data were converted to IUPAC codes and concatenated to FASTA format by variety. Fasta-formatted genotype data were used to generate a phylogenetic tree for the minimum core marker selection.

### Population structure analysis

Phylogenetic tree analysis was conducted using the UPGMA and NJ methods implemented in Molecular Evolutionary Genetics Analysis 7 (MEGA 7). PCA was performed using the R package SNPRelate and visualised using ggplot2. The population structure analysis was conducted using the fastStructure software, and the clusters were classified by the highest ancestry proportion of the model component. Boxplot analysis was conducted and plotted by grouping each cluster using ggplot, Duncan's new multiple range tests was conducted between the groups of clusters using the R package agricolae, and global ANOVA P-values were added using ggpubr. Different lowercase letters indicate significant differences between means at p < 0.05. Global P-values less than 0.05 were considered significant. The grouping of clusters was conducted based on significant differences between clusters.

## Supplementary Information

Supplementary Information 1.

Supplementary Information 2.

Supplementary Information 3.

Supplementary Information 4.

Supplementary Information 5.

Supplementary Information 6.

Supplementary Information 7.

Supplementary Information 8.

Supplementary Information 9.

## Data Availability

GBS reads of the 96 commercial cabbage varieties have been deposited in the National Center for Biotechnology Information (NCBI) with BioProject accession number PRJNA860655. The data is available at the following address: https://www.ncbi.nlm.nih.gov/bioproject/PRJNA860655. The genotypic data of the developed SNP markers for the 94 cabbages are included as supplementary information. All relevant data of this study are included in this published article and its supplementary information files.
