# Supplementary material for: Genome-wide analysis-based single nucleotide polymorphism marker sets to identify diverse genotypes in cabbage cultivars (Brassica oleracea var. capitata)
Source: Sci Rep. 2022 Nov 21;12:20030. doi: 10.1038/s41598-022-24477-y (PMC9681867; doi:10.1038/s41598-022-24477-y)
Supplement: Supplementary file 1 — Supplementary Information 1. [file 41598_2022_24477_MOESM1_ESM.pdf]

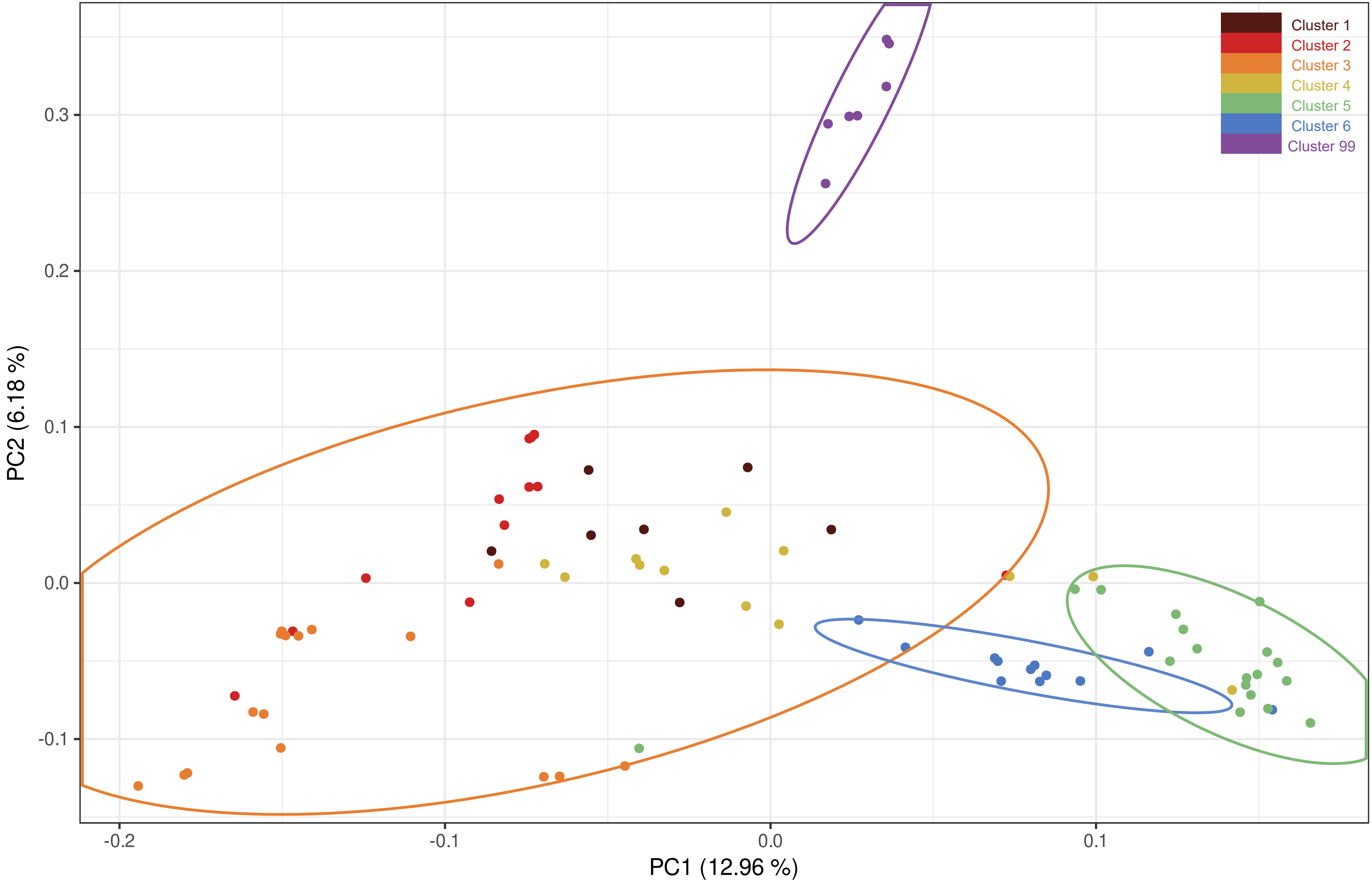

Supplementary Figure 1. Principal component analysis of 96 cabbage varieties genotyped with 26,301 of signle nucleotide polymorphisms (SNPs)
