# Supplementary material for: Genome-wide analysis-based single nucleotide polymorphism marker sets to identify diverse genotypes in cabbage cultivars (Brassica oleracea var. capitata)
Source: Sci Rep. 2022 Nov 21;12:20030. doi: 10.1038/s41598-022-24477-y (PMC9681867; doi:10.1038/s41598-022-24477-y)
Supplement: Supplementary file 2 — Supplementary Information 2. [file 41598_2022_24477_MOESM2_ESM.pdf]

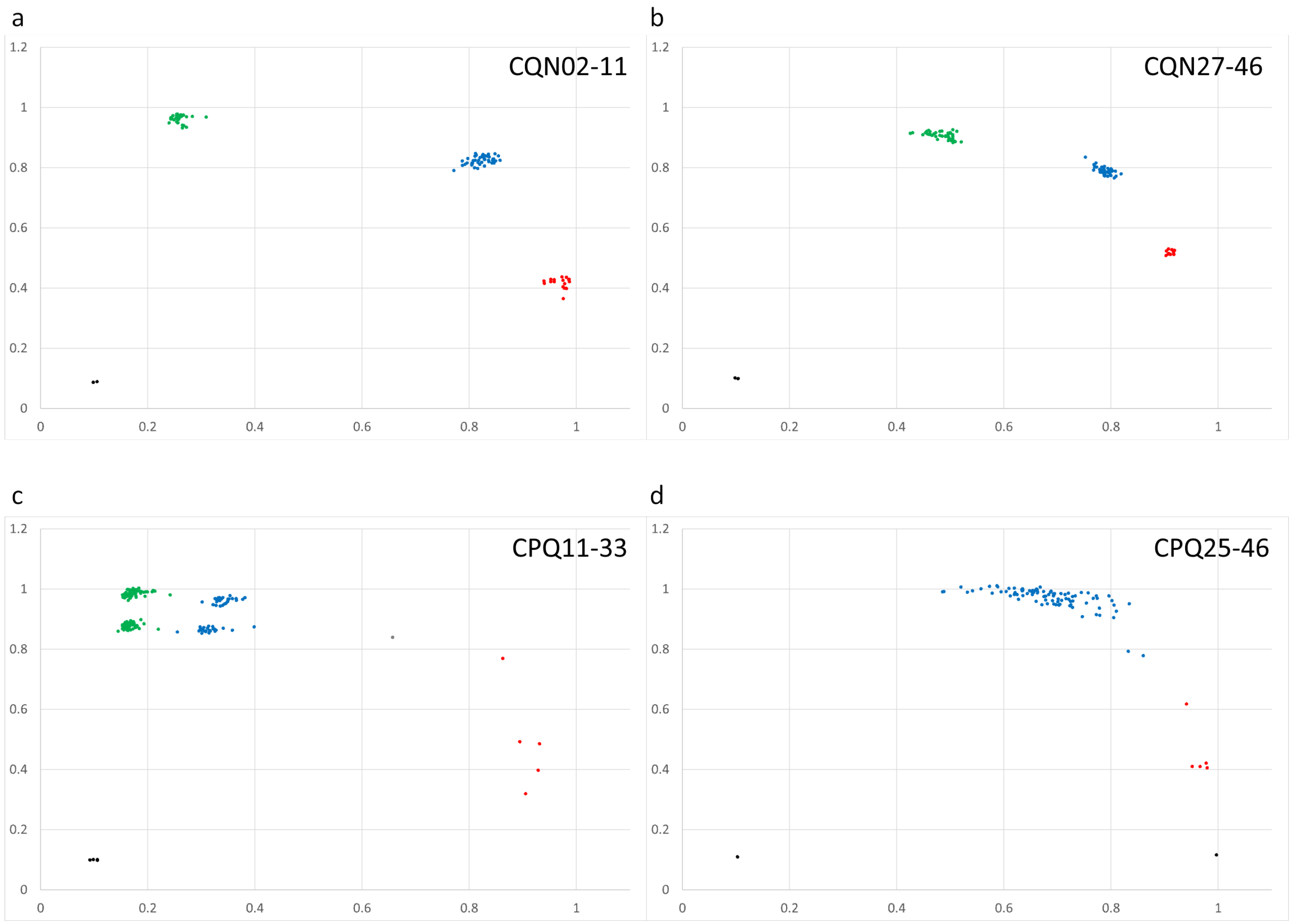

Supplementary Figure 2. Examples of scatter plots from the Fluidigm assay for 94 cabbage varieties. a. The genotype of CQN02-11, chosen as one of ten core markers, produced precise and accurate genotypes. b. The genotype of CQN27-46, chosen as 96 primer sets, produced precise and accurate genotypes. The genotype of CPQ11-33, excluded from 96 primer sets, produced precise but inaccurate genotypes. d. The genotype of CPQ25-46, excluded from 96 primer sets, produced inaccurate and imprecise genotypes. The X and Y axes indicate HEX and FAM florescence, respectively. Red and green dots indicate homozygous genotypes. Blue dots indicate heterozygous genotypes. Black dots indicate no template control, while grey dots indicate an unknown genotype.
